# Mother-Baby Friendly Philippines: Using Citizen Reporting to Improve Compliance to the International Code of Marketing of Breastmilk Substitutes

**DOI:** 10.9745/GHSP-D-21-00071

**Published:** 2022-02-28

**Authors:** Kate Reinsma, Alfred Jose C. Ballesteros, Rene Andrew A. Bucu, Teddy S. Dizon, Nathan John U. Jumalon, Lorelane C. Ramirez, Czarina Anne A. Villareiz, Carleneth San Valentin, Maria Rosario S. Vergeire

**Affiliations:** a World Vision International Technical Services Organization, Monrovia, CA, USA.; b Alliance for Improving Health Outcomes, Inc., Quezon City, Philippines.; c World Vision Development Foundation, Manila, Philippines.; d Undersecretary of Health, Health Regulation Team, Department of Health, Manila, Philippines.

## Abstract

**Introduction:**

In 1986, the Philippines was one of the first countries to pass national legislation on the International Code of Marketing of Breastmilk Substitutes in the form of Executive Order (EO) 51 and Republic Act (RA) 10028. While violations against the legislation and corresponding sanctions are clearly defined, infractions remain unreported or go unpunished. Enforcement of the laws remains a significant challenge as government capacities suffer from inadequate resources to regularly monitor breastfeeding-related law violations. To address these gaps, The Department of Health (Philippines) and the World Vision Development Foundation developed a reporting platform to enable citizen reporting of EO 51 and RA 10028 violations as part of the Mother-Baby Friendly Philippines (MBFP) initiative.

**Methods:**

Upon completion of the project, the Alliance for Improving Health Outcomes Inc. conducted 68 surveys and 24 key informant interviews and focus group discussions with individuals who participated in trainings on EO 51 and RA 10028, community health workers, representatives of local government units and hospitals involved in breastfeeding programs, and city health officials to document benefits, challenges, and lessons learned.

**Results:**

The intention and interest of consulted stakeholders to uphold the law by reporting violations through the platform indicate that citizen reporting can be harnessed as an effective tool for reporting violations. Nevertheless, multiple challenges remain in reporting and following up on Code violations.

**Discussion:**

The platform provided citizens with an opportunity to report violations, but, in reality, the status of action and feedback did not change. There is a need to strengthen implementation and enforcement at all levels of relevant national government agencies and improve feedback loops on reported violations.

## Abstract (precis)

Citizen reporting has the potential to improve compliance with the International Code of Marketing Breastmilk Substitutes. However, any investments made on improving citizen and reporting platforms must be matched with similar investments in streamlining government processes, transparency, and confidence-building across all stakeholders.

## Key Findings

- Any investments in improving platforms for reporting Code violations must simultaneously be matched with investments in government capacity to process reports and enforce Code violations.
- When working with multiple government agencies, a clear set of rules of procedure needs to be in place to guide the management of Code violations, provide feedback to citizen reporters on the status of their reports, and ensure that confidentiality is maintained at all levels.

## Key Implications

- Policy makers and program managers should routinely meet with interagency committees to discuss Code violation statistics and exceptional or troublesome cases and identify which government entities are doing specific actions to address the violations across various levels of the government.
- Program designers should include features such as gamification or a point system to keep users interested and engaged. This could even be as simple as ensuring that users receive feedback on the status of their reports to assure them that their submitted reports are pursued.

## INTRODUCTION

In 1981, the World Health Assembly adopted the International Code of Marketing of Breastmilk Substitutes^1^

*to contribute to the provision of safe and adequate nutrition for infants, by the protection and promotion of breastfeeding, and by ensuring the proper use of breastmilk substitutes, when these are necessary, based on adequate information and through appropriate marketing and distribution.*

The World Health Assembly also issued a series of subsequent resolutions clarifying and updating the International Code of Marketing of Breastmilk Substitutes, which must be read along with it and are referred to collectively as “the Code.” While evidence continues to show that breastfeeding provides essential, irreplaceable nutrition for a child’s growth, development, immunity and survival,^2^ aggressive marketing tactics by breastmilk substitute manufacturers pose a major obstacle to breastfeeding.^3^ Fueled by a rapidly growing infant formula market valued at over US$70.6 billion in 2019, breastmilk substitute manufacturers consistently unveil new products with new marketing techniques into new markets that violate the Code.^3^

According to the 2020 joint World Health Organization, UNICEF, and International Baby Food Action Network National Implementation of the International Code Status Report, 80 countries have a monitoring system to define sanctions for violations, but only 14 of those have a system that identifies who is responsible for monitoring compliance and requires that monitoring and enforcement be independent, transparent, and free from commercial influence.^4^ Programmatic experiences from Cambodia, Indonesia, Lao People’s Democratic Republic, Myanmar, Thailand, Vietnam, Timor-Leste, Burkina Faso, and Ethiopia indicate that developing a monitoring and enforcement system to regulate the actions of the breastmilk substitute industry and ensure compliance of health workers, media, and retailers, is quite challenging. It also suggests that developing tools for monitoring and reporting violations, including checklists and phone applications, and a reliable data management system to facilitate rapid follow-up by the government are key enablers to implementing a Code monitoring and enforcement system.^5^

In 1986, the Philippines was one of the first countries to pass national legislation on the International Code of Marketing of Breastmilk Substitutes. It also has one of the strongest legal frameworks to protect and promote breastfeeding in the form of the 2006 Revised Implementing Rules and Regulations of Executive Order (EO) 51 (The Milk Code of the Philippines) and Republic Act (RA) 10028 (Expanded Breastfeeding Promotion Act of 2009).^6^ Despite this, only 57.9% of Filipino children are exclusively breastfed and 54.1% are breastfed for 2 years.^7^ While violations against the legislation and corresponding sanctions are clearly defined, infractions remain unreported or go unpunished. Enforcement of the laws remains a major challenge as government capacities suffer from inadequate resources to regularly monitor breastfeeding-related law violations.^6^

In the Philippines, enforcement of breastfeeding-related laws remains a major challenge as government capacities suffer from inadequate resources to regularly monitor violations.

To address these gaps, the Philippines’ Department of Health (DOH), in cooperation with the World Vision Development Foundation, developed a reporting platform to enable citizen reporting of breastfeeding-related law violations as part of the Mother-Baby Friendly Philippines (MBFP) initiative. The idea of using citizen reporting came from successful initiatives against the marketing of soft drinks and cigarettes to children. Those initiatives were based on the notion that a public educated on the health risks of soda and tobacco consumption will lead to the mobilization of citizens advocating for change, forcing companies to adjust their advertising tactics.^8^ It was posited that a well-informed public educated on the importance of the breastfeeding-related laws would enable a public accountability system for monitoring and compliance. The Philippines was chosen to pilot this approach because it has one of the highest internet-use populations in the Association of South East Asian Nations (ASEAN) region, a high level of consumer engagement in social media, and high access to the internet via mobile/smartphones.^9^^,^^10^ This article summarizes the process, benefits, challenges, and lessons learned in developing a reporting platform to engage citizens in Code monitoring.

**Figure:**
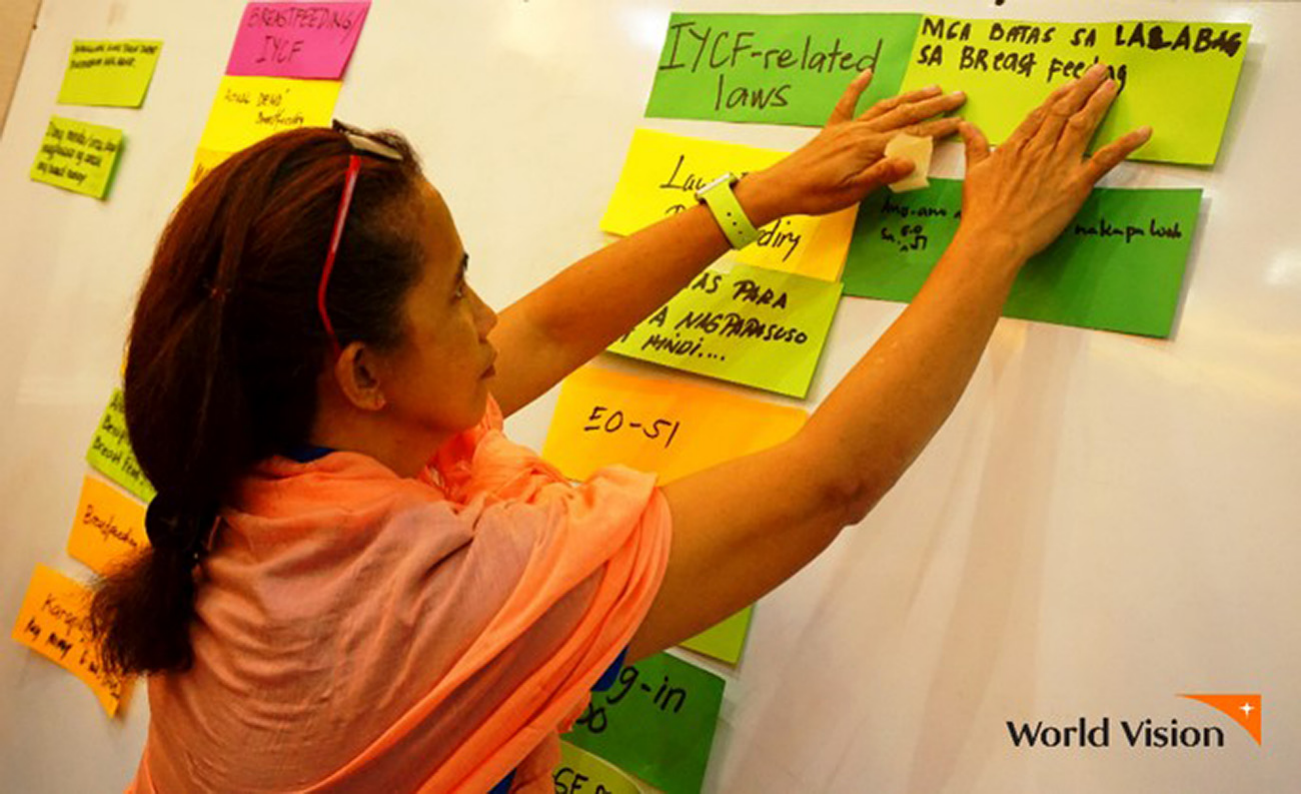
Training provided to health professionals that emphasized their need to know the Milk Code law beyond its title, how certain practices by formula companies impinge on their rights, and how to advocate for their rights by reporting violations. © 2018 Angela Pajares/World Vision Development Foundation

## THE TOOL AND TECHNOLOGY

The MBFP project was originally intended to improve the already existing Milk Code Monitoring website developed by the Philippine Food and Drug Administration (FDA) with funding from UNICEF. This website was used to report violations observed at health facilities, professional forums, and advertisements, including point of sales and packaging. Video clips, photos, and written reports were received through the site. The reports would then be reviewed and acted upon by the FDA. Since its launch in 2012, the website was described by both DOH and FDA to behave erratically or not be accessible. The website was falling behind technologically, and potential users reported difficulties in submitting the information required to process the violations, which compromised the validity and efficient processing of reports. Even more so, community members and local government units (LGUs) were generally unaware of the website’s existence, attributed to the lack of a communication strategy for dissemination and popularization of the website by DOH. Operation of the website ceased after UNICEF support discontinued in December 2016, citing domain hosting payments as the major challenge. In August 2017, it was decided that a new website be built and DOH take over hosting and management duties at the end of the MBFP project.

In cooperation with the Information Communication Technology partner ThinkBit and service provider Globe Telecom, the project developed a platform for reporting, processing, and resolving complaints of Milk Code violations via (1) a web-based reporting tool; (2) a mobile phone application (called Mother-Baby Friendly Philippines) available for both Android and iOS; and (3) short message service (SMS). To encourage reporting, SMS reporting was free of charge and did not consume phone credits. To circumvent lack of internet connectivity on the mobile applications, report details could be keyed in through the mobile application and submitted later when an internet connection became available.

The MBFP project developed a platform for reporting, processing, and resolving complaints of Milk Code violations using a website, a mobile phone application, and SMS.

The content of the MBFP platform was developed via an iterative process. Consultations were primarily held with representatives from DOH, FDA, and LGUs. On May 24–26, 2017, a technical working group workshop was held with representatives from DOH, FDA, LGUs, and ThinkBit to establish the data flow process, develop guidelines in reporting EO 51 and RA 10028 violations, and design the engineering requirements of the reporting platform. The initial version of the MBFP reporting platform was completed in time for its soft launch during the *Hakab Na!* (“Big Latch-On”) 2017 event to an audience of more than 2,000 mothers and at the ASEAN Breastfeeding Forum. The features of the website and the mobile application are outlined in the Table. These versions underwent user acceptance testing in October 2017 at one of the pilot sites in Quezon City, Philippines. Each channel, whether the website, the mobile application, or SMS, had respective steps for the submission of reports. Once reports were submitted, the platform sent 4 types of auto-generated messages to update the reporter on the status of the report: (1) if the report had been received; (2) if the report was determined to be valid; (3) if the report is under investigation; and (4) if the report has been resolved. The messages were sent to the contact information (email and/or mobile number) provided by the reporter.

**TABLE.** Features of the Mother-Baby Friendly Platform for Reporting Violations of the Milk Code

**Feature**	**Available On**	**Description**
Report violation	Website	Users enter data on date, place, and details of violation. Contact information (email and/or mobile phone number) is collected to allow reporters to receive updates on the status of their report. Information on the reporter (name, sector, age) are collected under conditions of confidentiality. Submission of the name of the reporter is optional. Reported violations submitted to the platform were filtered by WVDF and sent to DOH and FDA according to the type of violation. Reports are sent anonymously. Photo and video documentation can also be uploaded. Submitted photo and video documentation are collected as part of evidence and verified.
	Mobile application	Users had an outbox in case of slow or non-existent internet connections, allowing the submission of reports at a more appropriate time. Reporters could also view and track their reported violation statuses.
	SMS	Reports can also be sent to a designated number through SMS by providing the required details but uploading of supporting documentation is not possible.
News articles	Website and mobile application	WVDF-written news articles and updates on breastfeeding-related developments in the country (information on events, advocacy caravans, and updates on regulations and laws).
Infographics	Website and mobile application	Infographics formulated and published by WVDF with the approval of DOH designed to provide information on the rights of breastfeeding mothers, supporting policy documents, and the benefits of breastfeeding through what were intended to be attractive and more easily digestible graphical illustrations.
Videos	Website and mobile application	Videos produced and published by WVDF highlighting individual experiences of breastfeeding mothers, citing the benefits of breastfeeding for both mother and child.
Directory	Website	Users can view breastfeeding stations in the Philippines through a provided map. Upon identifying and clicking a chosen breastfeeding station, the user would be provided the address of the location and its accreditation status (i.e., whether it is an accredited Mother-Baby Friendly Hospital, accredited Mother-Baby Friendly Workplace, or has a certificate of commitment for Mother-Baby Friendly Program.)
	Mobile application	Users can suggest and upload breastfeeding station locations by marking a pin on the map, subject to approval of the administrator. The user will be required to upload an image of the location as well.
Policies and laws	Website and mobile application	Contains a database of policies and laws related to the MBFP project, including Executive Order 51 and its implementing rules and regulations and Regulation Act 10028 and its implementing rules and regulations. Users can also download an offline version of these laws.
Motherhood journal	Mobile application	Through the motherhood journal, a mother can track the activities of their baby/babies such as breastfeeding, diaper changes, and key development milestones and share these on social media. The section also provides tips on breastfeeding techniques and practices.
Other	Website and mobile application	Provides details of the MBFP project and the organizations involved, such as WVDF and DOH, with links to their respective websites. A frequently asked questions section is also provided for in-depth information.

Abbreviations: DOH, Department of Health; MBFP, Mother-Baby Friendly Philippines; SMS, short message service; WVDF, World Vision Development Foundation.

While the technology for the reporting platform was being developed, the MBFP project conducted various orientation and training activities for mothers and community health workers in 3 pilot areas: Manila, Malabon, and Quezon City. The pilot areas were chosen based on high population density, local health offices’ focus on strengthening breastfeeding and young child feeding, the high population percentage of and high underweight prevalence in children under the age of 5. These activities aimed to increase community awareness of the laws and to prepare for use of the reporting platform. The target audience included community members, government agencies, community health workers and volunteers, health professionals, breastfeeding advocates and advocacy groups reached through print (i.e., posters, booklets, flipcharts, brochures, and calendars) and audio-visual materials for use in workshops, conferences, and health promotion activities.

**Figure:**
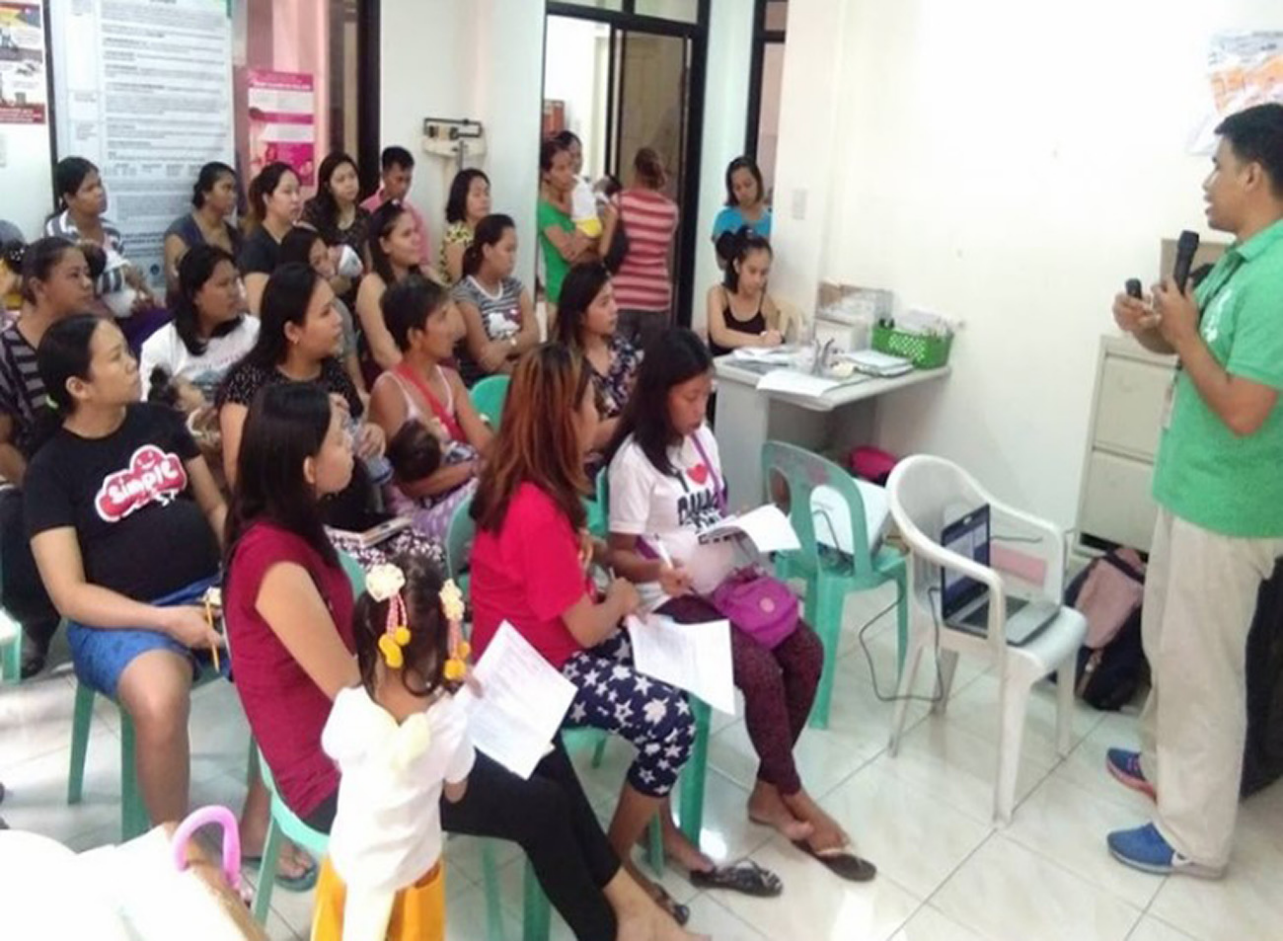
Community orientation on infant and young child feeding and related laws, as well as the Mother-Baby Friendly Philippines reporting platform. © 2018, Kristian Jebsen Bandong, World Vision Development Foundation

**FIGURE 1:**
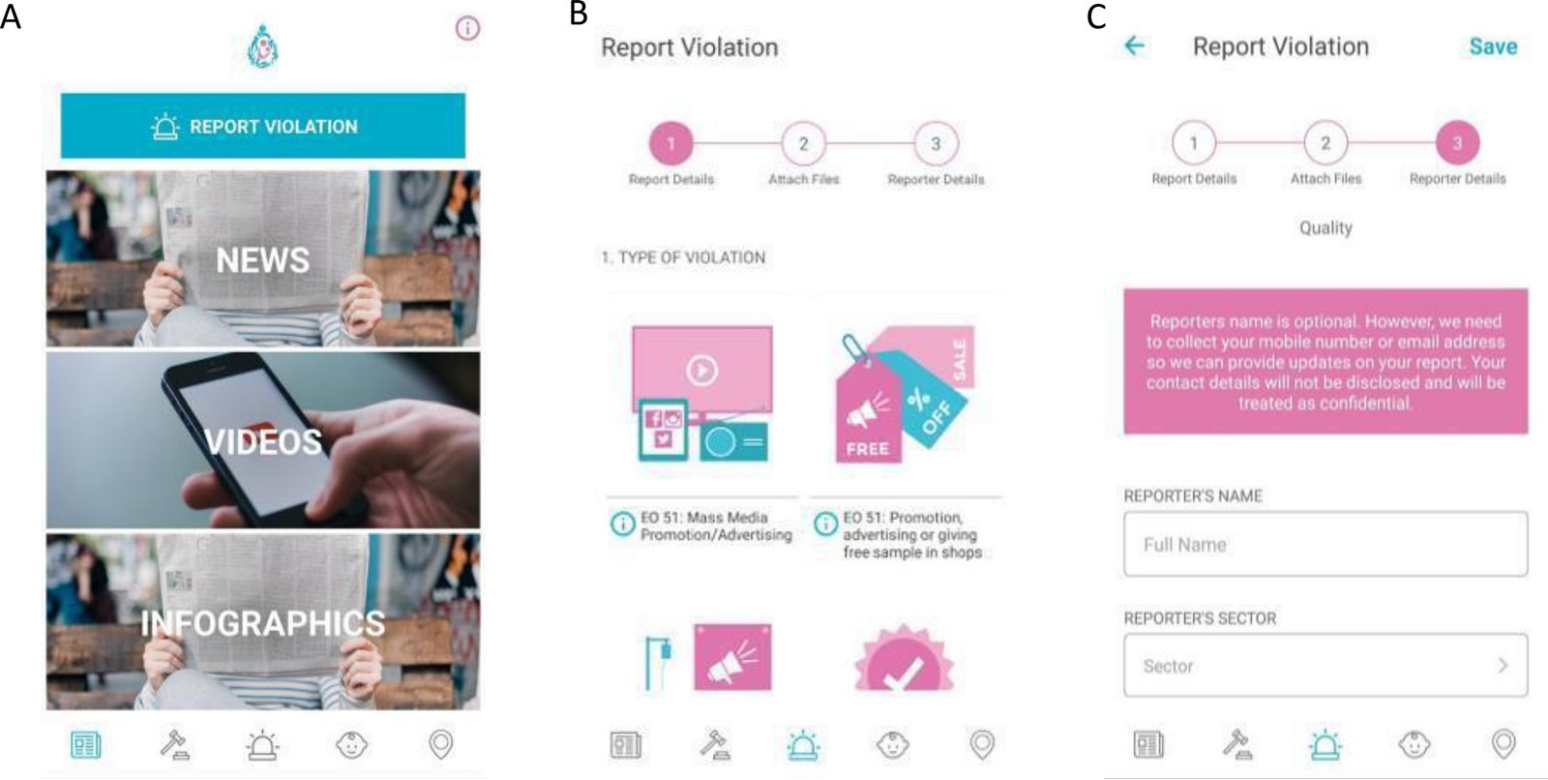
Screenshots of the Second Version of the Mother-Baby Friendly Philippines Mobile Application From left to right: (a) the dashboard or home screen, (b) the report form showcasing improved user interface with graphics and explanations, and (c) the violation reporting form.

After the reporting platform was launched, World Vision Development Foundation also conducted a series of internal consultations to expand and enhance the reporting platform functionality. Based on the results of the internal consultations, both the website and mobile application were updated to include early childhood care and development features, a growth monitoring chart, a user interface refresh, and Filipino translations (Figure 1).

**FIGURE 2:**
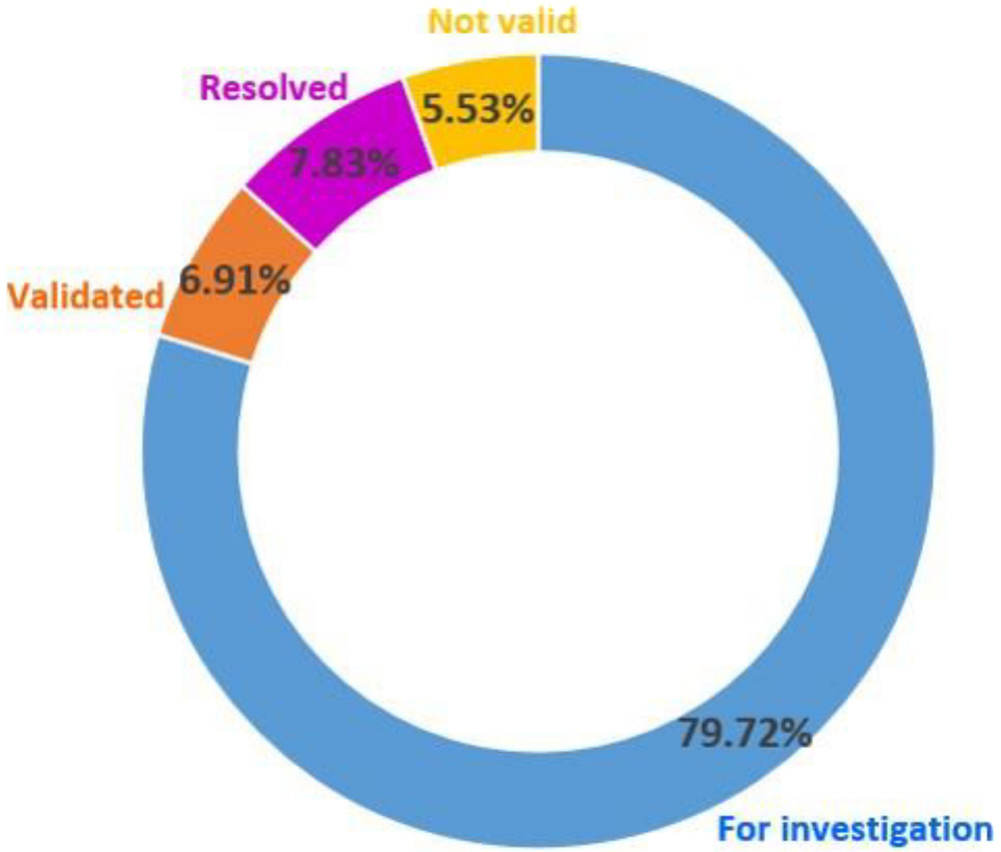
Status of Received Reports in Reporting Platform on Milk Code Violations, Philippines

Of the 3 reporting platforms developed (mobile app, website, and SMS), the mobile app (53.2%) and website (46.8%) were widely used to report violations. Of the 217 reports received, 205 were valid and 79.72% were transmitted to concerned government agencies for further investigation with 7.83% resolved (Figure 2). The erring parties were issued a show cause order by the FDA.

Of the 217 reports received, 205 were valid and 79.72% were transmitted to concerned government agencies for further investigation with 7.83% resolved.

### Methods

Upon completion of the project, Alliance for Improving Health Outcomes Inc. conducted a series of surveys, key informant interviews (KIIs), and focus group discussions (FGDs) to document benefits, challenges, and lessons learned in using a reporting platform to improve implementation of breastfeeding laws in the Philippines. The data were collected at the 3 pilot sites with the following respondents: (1) individuals who participated in training on EO 51 and RA 10028 and were instructed on the use of the MBFP reporting platform, held from 2017 to 2018; (2) trained community-level health workers; (3) representatives of LGU hospitals involved in breastfeeding programs; and (4) city health officials.

Data collection tools were designed with English and Filipino translations. Data from the survey, KIIs, and FGDs collected in Filipino were translated into English. The evaluation employed probability sampling for the survey, and nonprobability, purposive sampling for KIIs and FGDs identified by World Vision Development Foundation. A total of 68 respondents completed the survey, and 24 individuals participated in the FGDs and KIIs.

### Ethics Approval

The study protocol was granted ethics approval by the Asian Eye Institute Ethics Review Committee.

## RESULTS

### Benefits

**FIGURE 3:**
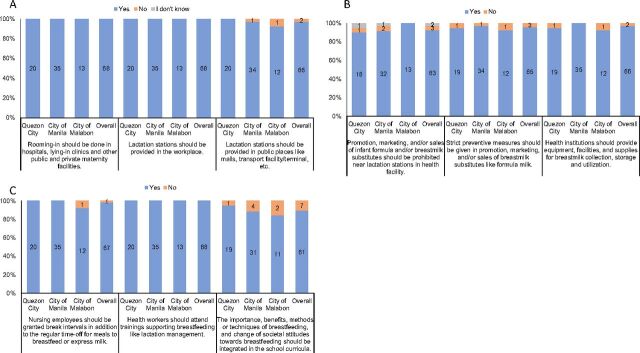
Awareness of Stipulations of the Breastfeeding Laws Among Respondents per Pilot Site, Philippines Abbreviations: MBFP, Mother-Baby Friendly Philippines; SMS, short message service.

As shown in Figure 3, results from the quantitative survey showed users of the platform had high awareness on activities that sought to promote or protect breastfeeding in their areas (e.g., rooming-in, prohibition of marketing of breastmilk substitutes, establishment of lactation stations, lactation breaks, human milk banks, milk-letting activities, and lactation management).

Participants in FGDs and KIIs claimed that the platform was very user-friendly, enabled more information to be accessible, and easily shared.

*We are able to report [violations] properly. We can give the right information. It is correct and clear. That is best when we can give them information. —*Community member, Manila

The platform also helped create an enabling environment to help LGUs underscore that violations should be taken seriously and to establish partnerships to support breastfeeding programs. LGU representatives also said that the influence of milk companies felt considerably less as compared to before. This was reiterated by hospital officials who expressed confidence that hospital personnel had been capacitated sufficiently for them to enforce implementation and prevent or manage violations at their level that are mostly bottle feeding in health facilities. Breastfeeding advocacy groups also observed that the quality of reports they had received over the 2 years had improved since reporters were able to identify a wider array of violations, possibly indicating a better grasp and feeling more empowered to report on violations.

*It helped somehow…moms are more empowered now. They know their rights. For example, if they are in the hospital and they give birth, and then their babies were not roomed in immediately…they will talk to the head nurse, contact them and tell them, “You know you’re violating RA 10028.” Sometimes they are separated for more than 24 hours, so they are violated. I believe it helps because apart from having websites this is very handy. (If) I’m in a hospital, maybe I see something, I’d go to the platform and see there’s a violation. They have a database thereof the laws. It’s easier.* —Breastfeeding Pinays advocacy group member

The platform also helped create an enabling environment to help LGUs underscore that violations should be taken seriously.

### Challenges

Participants from FGDs and KIIs identified major challenges as to why violations were not reported. Among all FGD and KII participants, none could sufficiently describe the steps on how to submit reports through the identified channels (website, mobile application, or SMS). Some participants also cited that they could not use the platform because they had no computers or smartphones to submit reports through the website or the mobile application. Participants also claimed that they could not submit reports because they did not have internet connectivity, mobile data, or mobile phone load. However, as noted previously, the platform allows for offline use such that reporters can encode details of the reported violations into the mobile application which can then be sent when internet connectivity is available. Sending reports through SMS was also free of charge. This suggests that users were not fully aware of the features of the platform to make reporting easier. There were also some participants who claimed that the platform is still too complicated and not user-friendly, despite ongoing revisions.

*We still need more training. We still do not comprehend how to report. —*Community member, Quezon City

Another major deterrent for reporting violations was the violation of privacy. The platform promised that reporters can submit their reports anonymously; however, there were cases when the reporters’ identities were divulged. City health officials who reported violations claimed that they were positively identified by their supervisors or by their coworkers. Members of breastfeeding advocacy groups also reported that they received harassment and threats through mobile phones while reports were being acted upon. Despite submitting reports and feedback that their privacy was breached, none of the complainants received any information or were made aware of the outcome on how their identities were divulged. As a result, some chose not to send anymore reports to preserve their relationships with coworkers due to fear of their identity being divulged again.

Although the platform promised that people could submit their reports anonymously, there were cases when reporters’ identities were divulged.

**FIGURE 4:**
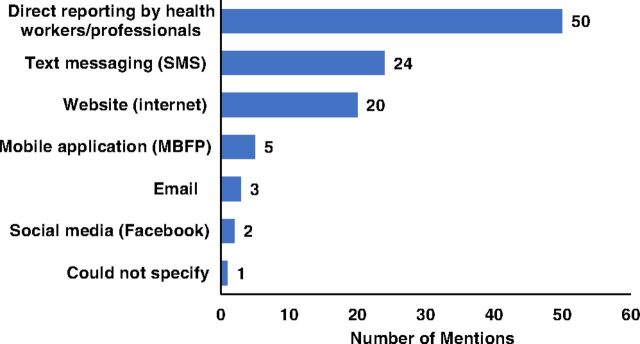
Possible Means of Reporting Violations as Identified by Survey Respondents, Philippines Abbreviations: MBFP, Mother-Baby Friendly Philippines; SMS, short message service.

Despite the increased level of awareness, the MBFP platform is not used or preferred when it comes to health workers reporting other health workers who committed violations, both at the hospital and community level. Among survey participants respondents most often mentioned direct reporting to health workers or professionals as the preferred method of reporting violations (Figure 4).

In the Philippines, there is the value of *pakikisama,* which simply means “getting along with others.” Since group harmony and unity are valued, health workers would opt not to report their colleagues through the reporting platform and would rather try to resolve the issue first at their level, whether through discussions or reprimands.

*Once I submit the report, the evidence is there. They say that there is confidentiality, and we trust that. But when our superior comes back to the health center, they say, “who has the wagging tongue and snitched?” It’s hard to tell a lie*. —Health care worker, Manila

Lastly, a major influence on not reporting violations is the lack of feedback on the processes and outcome of the reports submitted. Among the 68 survey participants, only 2 received any feedback. Among all FGD and KII participants, none reported knowing of any report that had been resolved nor the status of the outcome. According to the proposed process flows, reporters would receive autogenerated updates on their reports if it had been received/forwarded, if it was valid, and if it was resolved. However, not all would receive these autogenerated messages. The lack of feedback made reporters feel that their reports were not taken seriously or that they were not acted upon, similar to the old iteration of the reporting system. Also, it was reported by both managers of the MBFP platform (DOH) that there were no clear guidelines on how to update the status of reports.

*The [Interagency Committee] just keeps talking about the different reporting pathways, but we don’t understand that. We are not interested in that. We want to know where our reports go. What happens to the reports that we submit? They do not really understand their function. They do not want to commit or assume. Who will be the one to give out sanctions? Can the Interagency Committee remove licenses of violators?* —Manila City Health Office representative

## LESSONS LEARNED

Unfortunately, the original problems that beset the overall breastfeeding laws before revamping of the platform are still present, specifically the actual enforcement and implementation of the laws. At the beginning of this project, we thought that developing a reporting platform and encouraging citizen reporting would go a long way to improving Code compliance. We underestimated how important it was to have full participation of all relevant government authorities and stakeholders. While this project did seek to improve coordination with all relevant government agencies, developing the processes took almost a year and a half—much longer than we expected—and took a good portion of the 2-year project. Looking back, we wish we would have devoted more time and energy at the beginning of the project to improving government coordination and enforcement at the various tiers of the government. We recommend that program designers take this into consideration when designing similar projects.

Unfortunately, the original problems that beset the overall breastfeeding laws before revamping of the platform are still present, specifically the actual enforcement and implementation of the laws.

Some more of the specific lessons learned we learned from this project follow.

### Technology Is Not Always the Solution

While the ability to report Code violations on a website or through SMS may appear to be more convenient, this is not always the preferred method of the users. FGDs and KIIs showed that some people prefer a more traditional means of reporting, such as resolving the issue personally at their own level or through written notification to maintain the cultural value of pakikisama. Such methods must also be incorporated into a Code monitoring system.

### Ensure That Confidentiality Is Maintained at All Levels

It is important to guarantee the protection of the identities and privacy of the platform’s users to avoid further loss of confidence and trust. KIIs and FGDs showed that users were highly concerned about their privacy and confidentiality and their feelings of security corresponded to their perception of the platform as a viable avenue for reporting violations. The more they felt that their private lives and/or reputation were at risk, the less likely they were to use the platform. This would entail data privacy protection guidance for local and national government entities expected to act on violations.

Effectiveness of the reporting platform has to be demonstrated through active feedback from platform to the reporter and supported by periodically disseminating the statistics and taking actions based on the reports submitted. The lack of feedback gave reporters the feeling of uncertainty about the treatment of their reports, either they were not taken seriously or were abandoned. The lack of feedback also led to the perception that the interagency committee, the body responsible for acting on the violations, was not performing its implementation functions.

### Establish a Rules of Procedure to Guide the Management of Violations

Since there was confusion between the roles of DOH and interagency committee as the 2 implementers of EO 51, there is a need to clearly identify which government entities are doing specific actions to address violations. The responsibilities should be assigned to dedicated personnel as mandated tasks instead of being additional work on top of existing responsibilities. Setting up a Rules of Procedure takes a significant amount of time and should be accounted for in future projects.

## CONCLUSION

Evaluation of the MBFP project reveals that there is a general awareness of the breastfeeding laws and the existence of the reporting platform. The intention and interest of consulted stakeholders to uphold the law by reporting violations through the platform indicate that citizen reporting can be harnessed as an effective tool for reporting violations. However, reporting mechanisms need to be made appropriate and accessible for all social classes and allow for multiple forms of submission (phone calls, SMS, website, or paper) for reporting Code violations.

While the platform provided an opportunity for citizens to do their part by submitting reports of violations, additional efforts are needed to strengthen the implementation and enforcement at all levels of relevant national government agencies and improving the feedback loop and status updates on reports enacted. To achieve this, we suggest at least quarterly meetings with an Interagency Committee to discuss Code violation statistics and exceptional or troublesome cases and identify which government entities are doing specific actions to address the violations across various levels of the government.

Finally, to keep users engaged in using the reporting platform, we suggest using features such as gamification or a point system. During this project, we sought to engage users by making the mobile application useful for them by including a motherhood journal, an early childhood development checklist, and relevant information about breastfeeding, but more could be done to encourage citizen reporting of Code violations.

## Notes

**Peer Reviewed**

**First published online:** January 25, 2022.
